# The aryl hydrocarbon receptor regulates lipid mediator production in alveolar macrophages

**DOI:** 10.3389/fimmu.2023.1157373

**Published:** 2023-04-04

**Authors:** Ann-Marie Maier, Karsten Huth, Francesca Alessandrini, Fiona Henkel, Benjamin Schnautz, Anela Arifovic, Fabien Riols, Mark Haid, Anja Koegler, Katrin Sameith, Carsten B. Schmidt-Weber, Julia Esser-von-Bieren, Caspar Ohnmacht

**Affiliations:** ^1^ Center of Allergy and Environment (ZAUM), Technical University of Munich and Helmholtz Center Munich, Research Center for Environmental Health, Neuherberg, Germany; ^2^ Metabolomics and Proteomics Core, Helmholtz Center Munich, Research Center for Environmental Health, Neuherberg, Germany; ^3^ DRESDEN-concept Genome Center, Technology Platform at the Center for Molecular and Cellular Bioengineering (CMCB), Technische Universität Dresden, Dresden, Germany; ^4^ Member of the German Center of Lung Research (DZL), Partner Site Munich, Munich, Germany; ^5^ Department of Immunobiology, University of Lausanne, Epalinges, Switzerland

**Keywords:** macrophage, aryl hydrocarbon receptor, eicosanoids, leukotriene, prostaglandin

## Abstract

Allergic inflammation of the airways such as allergic asthma is a major health problem with growing incidence world-wide. One cardinal feature in severe type 2-dominated airway inflammation is the release of lipid mediators of the eicosanoid family that can either promote or dampen allergic inflammation. Macrophages are key producers of prostaglandins and leukotrienes which play diverse roles in allergic airway inflammation and thus require tight control. Using RNA- and ATAC-sequencing, liquid chromatography coupled to mass spectrometry (LC-MS/MS), enzyme immunoassays (EIA), gene expression analysis and *in vivo* models, we show that the aryl hydrocarbon receptor (AhR) contributes to this control *via* transcriptional regulation of lipid mediator synthesis enzymes in bone marrow-derived as well as in primary alveolar macrophages. In the absence or inhibition of AhR activity, multiple genes of both the prostaglandin and the leukotriene pathway were downregulated, resulting in lower synthesis of prostanoids, such as prostaglandin E2 (PGE_2_), and cysteinyl leukotrienes, e.g., Leukotriene C4 (LTC_4_). These AhR-dependent genes include *PTGS1* encoding for the enzyme cyclooxygenase 1 (COX1) and *ALOX5* encoding for the arachidonate 5-lipoxygenase (5-LO) both of which major upstream regulators of the prostanoid and leukotriene pathway, respectively. This regulation is independent of the activation stimulus and partially also detectable in unstimulated macrophages suggesting an important role of basal AhR activity for eicosanoid production in steady state macrophages. Lastly, we demonstrate that AhR deficiency in hematopoietic but not epithelial cells aggravates house dust mite induced allergic airway inflammation. These results suggest an essential role for AhR-dependent eicosanoid regulation in macrophages during homeostasis and inflammation.

## Introduction

Allergic airway inflammation including asthma is a major health burden with growing world-wide incidence. Treatment options are limited and include anti-histaminic drugs, general immune suppression by corticosteroids or targeting of type-2 cytokines by monoclonal antibodies. Eicosanoids are bioactive metabolites derived from the polyunsaturated fatty acid arachidonic acid that play important pro- but also anti-inflammatory roles in severe type-2 inflammation including allergic asthma (1). Current therapies targeting eicosanoid pathways have failed to provide a major advance in the therapy of asthma, owing to a limited understanding of the regulatory mechanisms that control these complex mediators. Arachidonic acid can be metabolized to generate bioactive eicosanoids *via* two distinct pathways: prostanoids are formed *via* the activity of two isozymes (cyclooxygenase 1 (COX-1) and cyclooxygenase 2 (COX-2), encoded by *PTGS1* and *PTGS2*, respectively) ultimately resulting in the generation of thromboxane and prostaglandins (PGs) including prostaglandin E_2_ (PGE_2_) and prostacyclin (PGI_2_). Importantly, COX1 is expressed constitutively in many cell types, while COX2 expression can be induced by different stimuli including allergens (2, 3). Both enzymes catalyze the formation of prostaglandin H_2_ (PGH_2_) which can then be further metabolized by specific enzymes to PGE_2_, prostaglandin D_2_ (PGD_2_), prostaglandin F_2α_ (PGF_2α_), PGI_2_ and thromboxanes A_2_ (TXA_2_) and B_2_ (TXB_2_), collectively known as prostanoids. Alternatively, the activity of 5-lipoxygenase (5-LO, encoded by *ALOX5)* catalyzes the formation of leukotrienes (LTs) such as the pro-inflammatory leukotriene B_4_ (LTB_4_), C_4_ (LTC_4_) and D_4_ (LTD_4_). While LTs and PGD_2_ are important drivers of type-2 inflammation (4–6), PGE_2_ and PGI_2_ have anti-inflammatory functions and can dampen allergic inflammatory responses (7–9). Thus, the eicosanoid system represents an important layer of immune regulation in type 2 immunity dominated disorders (10).

Myeloid cell types such as macrophages represent a major cell type involved in the generation of bioactive LTs and prostanoids (11, 12). In recent years, macrophages were shown to constitute a highly plastic and heterogenous cell population that can adapt to various microenvironments by adopting metabolically and functionally different cellular states. Specifically, type-2 cytokines such as interleukin-4 but also other environmental factors trigger the expression of enzymes to foster LT and prostanoid biosynthesis (13). However, there is currently a lack of understanding which transcription factors are enabling and regulating eicosanoid synthesis and function in macrophages.

The aryl hydrocarbon receptor (AhR) is a ligand activated transcription factor that is present in the cytosol and upon binding to its ligands can translocate to the nucleus and induce gene transcription. AhR ligands include aromatic hydrocarbons mostly derived from external sources but also endogenous ligands of the tryptophan catabolism such as indole derivatives (14). Depending on structural differences, AhR ligands may act as agonists or competitive antagonists probably contributing to the diverse AhR functions ranging from detoxification, maintaining stemness to cellular differentiation processes (15). We have previously demonstrated an important role for AhR in the regulation of allergic airway inflammation (AAI) in a murine house dust mite (HDM) model (16). However, which pathways are dysregulated in the absence of AhR remains unclear. Based on previous reports suggesting a role for AhR in the regulation of eicosanoids in an epithelial cell line (17), we investigated in this study whether the AhR contributes to the regulation of eicosanoids in alveolar macrophages.

Our results demonstrate that AhR serves as a transcriptional regulator for a set of genes in alveolar or alveolar-like macrophages (COX-1, 5-LO and leukotriene C4 synthase (LTC4S), encoded by (*PTGS1*, *ALOX5* and *LTC4S*)). Moreover, production of eicosanoids including all cysteinyl leukotrienes (cysLTs), LTC_4_, LTD_4_ and LTE_4_, and PGE_2_ was impaired in AhR insensitive macrophages induced by either chemical inhibition or genetic AhR deficiency. This defect in eicosanoid production was readily detectable at steady state and maintained across a set of different stimuli suggesting a central role for the AhR in the regulation of basic eicosanoid biosynthesis capacity of macrophages. Lastly, mice with hematopoietic- but not epithelial-specific AhR deficiency showed a more severe inflammation in an HDM model of allergic airway inflammation including cellular infiltration to the bronchoalveolar fluid. Altogether, our results suggest a central role for the AhR in alveolar macrophages’ ability to metabolize arachidonic acid into functionally active LTs and PGs which might be instrumental to prevent exaggerated type 2 inflammation in the lung.

## Materials and methods

### Animals

C57/B6/J wildtype (WT), AhR^KO/KO^ (18) (B6.129-*Ahr^tm1Bra^*/J, Jax strain number 002831), Vav^iCre^ (19) (B6.Cg-*Commd10^Tg(Vav1-icre)A2Kio^*/J, Jax strain number 008610), LysM^Cre^ (20) (B6.129P2-*Lyz2^tm1(cre)Ifo^*/J, Jax strain number 004781), Scgb1a1^CreERT2^ (21) (B6N.129S6(Cg)-*Scgb1a1^tm1(cre/ERT)Blh^*/J, Jax strain number 016225) and AhR^flox^ (22) (*Ahr^tm3.1Bra^*/J, Jax strain number 006203) mice were bred and kept under specific pathogen-free conditions in individually ventilated cages at the central animal facility (CF-LAS) of Helmholtz Center Munich. To achieve cell-specific deletion of AhR, mice carrying a loxP-flanked exon of the AhR gene (AhR^flox/flox^) were either crossed with mice expressing the Cre recombinase under control of the Vav promoter (Vav^iCre^) or with mice expressing a Cre recombinase under control of the lysozyme promotor (LysM^Cre^). For cell-specific deletion of AhR in lung airway epithelial cells, AhR^flox^ mice were bred with Scgb1a1^CreERT2^ mice and offsprings were treated with a single administration of tamoxifen at three weeks of age (5 mg intragastrically) and were then weaned on a tamoxifen-containing diet (500 mg/kg tamoxifen citrate (Ssniff special diets, Soest, Germany)) throughout the experiment. Both male and female offspring mice between 7-14 weeks were used. All experiments were performed with age- and sex-matched mice, under guidelines of the European Convention for Animal Care and Use of Laboratory Animals and were approved by local ethics committee and government authorities (ROB-55.2-2532.Vet_02-18-94 and ROB-55.2-2532.Vet_02-17-222).

### Differentiation of bone marrow-derived macrophages

BMDMs of wildtype (WT), AhR^KO/KO^, Vav^iCre^ x AhR^flox/flox^ and LysM^Cre^ x AhR^flox/flox^ mice were differentiated as described with minor modifications to better mimic alveolar-like macrophages (23, 24). Briefly, bone marrow was flushed from femur and tibia, filtered with cell strainers (70 µm) and 1.5 x 10^7^ cells were cultured in a TC cell culture flask (T-75, Sarstedt, Nümbrecht, Germany) in 15 ml RPMI-1640 medium (ThermoFisher Scientific) containing 10 ng/mL GM-CSF (Peprotech) and 2 ng/mL TGF-ß1 (Peprotech) to drive differentiation of alveolar-like BMDMs. After three days 50% of the medium was exchanged with new medium containing 20 ng/ml GM-CSF and 4 ng/ml hTGF-ß1. On day 6 the cells were stimulated with 10 µg/ml HDM extract (Citeq BV), 97 EU/ml LPS (L4130, Sigma Aldrich), 10 µg/ml *Ascaris suum* (A.S.) L3 extract, 10 µg/ml *Heligmosomoides polygyrus bakeri* (Hpb) L3 extract or apoptotic cells. After 24 h of stimulation 5 µM Ca^2+^-ionophore A23187 (Sigma Aldrich) was added for 10 min to ensure full release of lipid mediators. Prior analysis, supernatants were stored at −80°C, undiluted for ELISA analysis or 1:1 diluted with mass spectrometry grade methanol for LC-MS/MS analysis. Cells were lysed with RNA lysis buffer (Zymo Research) and stored at -80°C until RNA isolation. When mentioned, cells were treated with 10 µM AhR inhibitor CH-223191 (Sigma Aldrich, stock solution at 100 mM in DMSO) either from the start of the culture (day 0) or at day 6 prior BMDM stimulation until the end of the stimulation.

### RNA-sequencing

BMDMs (differentiated and treated as described) were lysed in RLT Plus Buffer (Qiagen, Hilden, Germany) containing β-ME and stored at -80°C until RNA isolation. RNA isolation was performed using the RNeasy PLUS Mini Kit (Qiagen) according to the manufacturer’s instructions.

mRNA was isolated from 70 ng total RNA by poly-dT enrichment using the NEBNext Poly(A) mRNA Magnetic Isolation Module (NEB, Ipswich, MA, USA) according to the manufacturer’s instructions. Samples were then directly subjected to the workflow for strand-specific RNA-Seq library preparation (Ultra II Directional RNA Library Prep, NEB). Custom adaptors were used for ligation (Adaptor-Oligo 1: 5’-ACA CTC TTT CCC TAC ACG ACG CTC TTC CGA TCT-3’, Adaptor-Oligo 2: 5’-P-GAT CGG AAG AGC ACA CGT CTG AAC TCC AGT CAC-3’) and adapter dimers were depleted by XP bead purification (Beckman Coulter, Brea, CA, USA). Indexing (unique dual index) was done during PCR enrichment (14 cycles, 65°C) using custom amplification primers carrying the same sequence for i7 and i5 index (Primer 1: AAT GAT ACG GCG ACC ACC GAG ATC TAC AC NNNNNNNN ACA TCT TTC CCT ACA CGA CGC TCT TCC GAT CT, Primer 2: CAA GCA GAA GAC GGC ATA CGA GAT NNNNNNNN GTG ACT GGA GTT CAG ACG TGT GCT CTT CCG ATC T). Libraries were quantified using the Fragment Analyzer (Agilent, Santa Clara, CA, USA) and equimolarly pooled and sequenced with paired-end 100bp reads on the NovaSeq 6000, to an average depth of 50 million reads each. The raw data is available at the NCBI’s Gene Expression Omnibus database and accessible through GEO Series accession number GSE221093 (https://www.ncbi.nlm.nih.gov/geo/query/acc.cgi?acc=GSE221093).

### RNA-sequencing analysis

RNA-seq bioinformatic analysis was done using an in-house analysis pipeline of the DRESDEN-concept Genome Center. Basic quality control of the raw sequencing reads was performed using FastQC (https://www.bioinformatics.babraham.ac.uk/projects/fastqc/) and RNA-SeQC (version 1.1.8) (25). Reads were aligned to the mm10 reference genome using GSNAP (version 2020-12-16) (26); the Ensembl gene annotation version 98 was used for splice site detection. Uniquely aligned reads per gene were counted using featureCounts (version 2.0.1) (27) and the respective Ensembl annotation. Normalization of raw read counts based on library size, and testing for differential gene expression between conditions was performed using the DESeq2 R package (version 1.30.1) (28) and R version 3.5.1. Genes with an absolute fold-change ≥ 2 and an adjusted *p*-value ≤ 0.05 are considered as differentially expressed genes (DEGs).

### ATAC-sequencing

Libraries were prepared using the Nextera DNA library Prep Kit (Illumina, San Diego, CA, USA) as previously described (29). Briefly, 5x10^4^ BMDMs (differentiated and treated as described above) were washed in PBS (ThermoFisher Scientific) and then lysed in 10 mM Tris-HCl, pH 7.4, 10 mM NaCl, 3 mM MgCl_2_ and 0.1% Igepal CA-630 (all Sigma Aldrich). After centrifugation the nuclei were resuspended in transposition mix (25 mL TD (2x reaction buffer (Illumina, San Diego, CA, USA)), 2.5 mL TDE1 (Nextera Tn5 Transposase (Illumina)) and 22.5 mL nuclease-free water), incubated for 30 min at 37°C. The tagmented DNA was purified with the MinElute PCR Purification Kit (Qiagen).

A total of 10 µl of purified tagmented DNA was indexed and pre-amplified for initial 5 PCR cycles with 1x KAPA HiFi HotStart Readymix (Roche, Basel, Switzerland) and 100 nM unique dual index P5 and P7 primers compatible with Illumina Nextera DNA barcoding, under the following PCR conditions: 72°C for 5 min, 98°C for 30 s, thermocycling for 5 cycles at 98°C for 10 s, 63°C for 30 s and 72°C for 1 min. To avoid saturation and potential biases in library amplification (see (29)), a qPCR (LightCycler 480 (Roche)) was performed with 1 µl of the pre-amplified material to determine the remaining PCR cycle numbers. Purification and double-sided size selection of amplified libraries was done with XP beads (Beckmann Coulter; starting with a 1.3x volume of XP bead purification, followed by a 0.6x/1.3x double-sided size selection). Libraries were quantified using the Fragment Analyzer (Agilent) and equimolarly pooled and sequenced with paired-end 100 bp reads on the NovaSeq 6000 (Illumina), to an average depth of 50 million reads each. The raw data is available at the NCBI’s Gene Expression Omnibus database and accessible through GEO Series accession number GSE221144 (https://www.ncbi.nlm.nih.gov/geo/query/acc.cgi?acc=GSE221144).

### ATAC-sequencing analysis

ATAC-seq bioinformatics analysis was done using the nf-core atacseq pipeline (30) (with “–*genome mm10 -r 1.2.1*”, and default settings otherwise). The pipeline is very comprehensive and automatically runs all required steps on a library-level, including (but not limited to) raw read QC, adapter trimming, alignment, filtering, generation of normalized coverage tracks, peak calling, and peak annotation based on the closest neighboring gene. Finally, a consensus peak set is created across all libraries, and differential accessibility analysis performed. Peaks with an absolute fold-change ≥ 1.5 and an adjusted *p*-value ≤ 0.05 are considered as differentially accessible regions (DARs).

### Combined analysis of RNA-seq and ATAC-seq data sets

**Figure 1:**
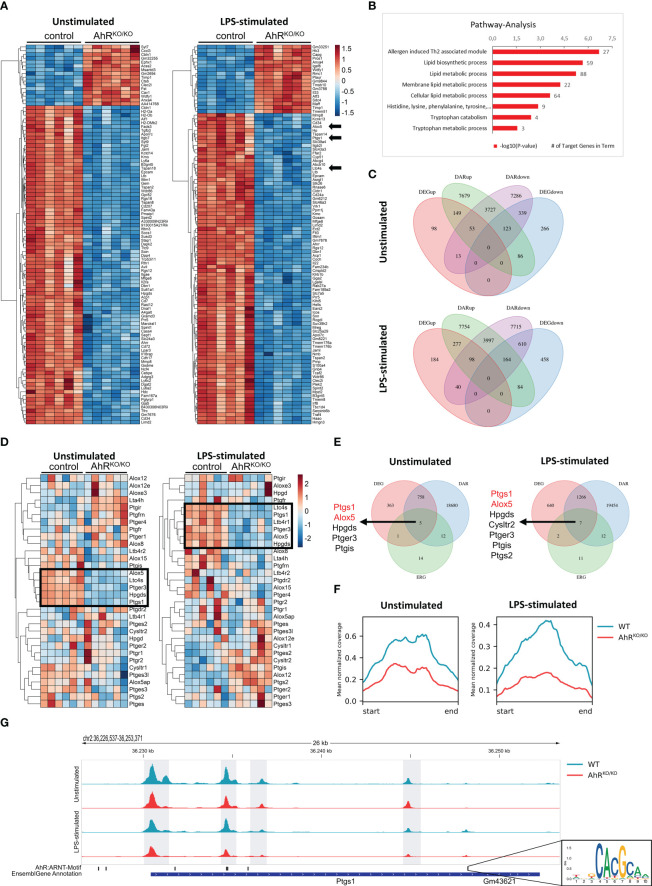
Bone marrow-derived macrophages (BMDMs) reveal AhR-dependent differential accessibility and gene expression of genes involved in eicosanoid biosynthesis. **(A)** Heatmap of the top100 differentially expressed genes (DEGs) in unstimulated (left) and LPS-stimulated (right) BMDMs of either wildtype (control) or AhR-deficient (AhR^KO/KO^) animals. **(B)** KEGG pathway analysis of DEGs from LPS-stimulated BMDMs (selection). Shown are the -log10 p value. Numbers indicate target genes in pathway. **(C)** Venn diagrams of intersections of up- and downregulated DEGs and genes linked with differentially accessible chromatin (DAR) in unstimulated (upper plot) and LPS-stimulated BMDMs (lower plot). **(D)** Heatmap of eicosanoid related genes (ERG) expression of either unstimulated (left) or LPS-stimulated (right) WT and AhR^KO/KO^ BMDMs. **(E)** Venn diagrams of DEGs, DARs and ERGs of either unstimulated (upper plot) or LPS-stimulated (lower plot) BMDMs. Genes in intersection are presented left to Venn diagrams. **(F)** Mean normalized coverage of all downregulated DARs of the intersection identified in **(E)**. **(G)** ATAC seq track of the Ptgs1 gene locus in WT and AhR^KO/KO^ of unstimulated or LPS-stimulated BMDMs. AhR/Arnt binding motif (inlet) of this region in the mouse genome is shown in the lower track. Data are derived from N = 6 animals per group from one experiment.

To select promising candidate peaks and genes, we filtered for DARs that are associated to DEGs and eicosanoid related genes (ERGs, see **Figure 1E**). The mean normalized ATAC-seq coverage across these DARs was visualized with deepTools (**Figure 1F**, version 3.5.1 (31)). The Arnt : Ahr binding motif (TBGCACGCAA) was retrieved from Homer (http://homer.ucsd.edu/homer/motif/HomerMotifDB/homerResults.html) and scanned in the mouse mm10 reference genome with Homer scanMotifGenomeWide.pl (version 4.11 (32)).

### Isolation of alveolar macrophages

Alveolar macrophages were isolated and cultured as previously described (33). Briefly, WT C57BL/6J and AhR^KO/KO^ mice were sacrificed and lungs were flushed 9 times with 1 mL of warm PBS (ThermoFisher Scientific, Rockford, IL, USA) containing 0,5% FBS (Sigma Aldrich, St Louis, MO, USA) and 2 mM EDTA (ThermoFisher Scientific). The collected BAL fluid (BALF) was added to a collection tube containing 3 mL RPMI 1640 (ThermoFisher Scientific) with 10% FBS, 1% GlutaMAX (ThermoFisher Scientific), 1% Pyruvate (ThermoFisher Scientific), 1% Penicillin/Streptomycin (ThermoFisher Scientific). After centrifugation, 1.5x10^5^ cells per well were plated in a 96-well plate containing 200 µL of RPMI 1640 with 10% FBS, 1% Penicillin/Streptomycin, 1% GlutaMAX, 1% Pyruvate, 10 ng/mL GM-CSF (Peprotech, Cranbury, NJ, USA) and incubated at 37°C and 5% CO_2_. On day 3 of incubation the medium was exchanged and the cells were stimulated with 10 µg/ml HDM extract (Citeq BV, Groningen, NL) or 97 EU/mL lipopolysaccharide (LPS) (Sigma Aldrich). The amount of LPS was chosen according to the LPS concentration present in the HDM extract. After 24 h of stimulation 5 µM Ca^2+^-ionophore A23187 (Sigma Aldrich) was added for 10 min for complete release of lipid mediators. Supernatants were stored at −80°C for ELISA analysis and cells were lysed with RNA lysis buffer (Zymo Research, Irvine, CA, USA) and stored at -80° until RNA isolation.

### RNA extraction and real-time PCR

Alveolar macrophages or BMDMs were lysed in lysis buffer (Zymo Research) followed by RNA isolation using the Quick-RNA MicroPrep Kit (Zymo Research) according to the manufacturer’s instructions. Isolated RNA was reverse transcribed using a cDNA synthesis kit (Thermo Fisher Scientific) according to the manufacturer’s instructions. Realtime/qPCR was performed using SYBR Green Master Mix (Roche) according to the manufacturer’s instructions. Transcripts were normalized to two housekeeping genes (GAPDH and ß-actin). The relative expression was calculated with the 2^−ΔCT^ method. Primer sequences are listed in **Supplementary Table 1**.

### Quantification of eicosanoids

BMDM supernatants were prepared and analyzed for eicosanoids by LC-MS/MS as previously published (12). Cysteinyl leukotrienes (cysLTs) and prostaglandin E2 (PGE_2_) were quantified using enzyme-linked immunosorbent assay (ELISA) kits (Cayman, Ann Arbor, MI, USA) according to manufacturer’s instructions.

### Western blot

Protein concentration of cell lysate received from bone marrow-derived macrophages (differentiated and treated as described above) was determined by Pierce™ BCA protein assay kit (Thermo Scientific) and diluted in RIPA lysis buffer (ThermoFisher Scientific) to load 45 µg/lane for separation by SDS-PAGE at 120 V (constant) for 1 h on a 10% Tris-Tricine gel. After transferring onto a nitrocellulose membrane (Carl Roth, Karlsruhe, Germany) (1 h at 100mA) blots were blocked for 1 h with 4% (w/v) nonfat dry milk powder (AppliChem, Darmstadt, Germany) in Tris-buffered saline with 0.1% Tween-20 (TBST) (Sigma Aldrich). Membranes were then incubated overnight at 4°C with a diluted polyclonal rabbit antibody against LTC4S (ab91507, dilution 1:300 (Abcam, Cambridge, UK)), 5-LO (a kind gift by Olof Rådmark, Karolinska Institute Stockholm, dilution 1:500) or β-actin (PA1-183, dilution 1:500 (Invitrogen, Carlsbad, CA, USA) in 2% nonfat dry milk powder in TBST (Sigma Aldrich). After washing the membrane 5 times with TBST (Sigma Aldrich), bound antibodies were detected by incubating an alkaline phosphatase-conjugated secondary polyclonal goat anti-rabbit IgG antibody (SAB3700854, dilution 1:5000 (Sigma Aldrich)) in 2% (w/v) nonfat dry milk powder in TBST (Sigma Aldrich) for 1 h at room temperature. The membrane was washed three times with TBST (Sigma Aldrich) and alkaline phosphatase was detected and visualized with BCIP/NBT (AppliChem) substrate. After development the reaction was stopped with ddH_2_O and the membranes were dried and scanned.

### Mouse model of allergic airway inflammation

Mice were sensitized by intranasal (i.n.) instillation of 1 µg house dust mite extract (HDM) of *Dermatophagoides farinae* (Citeq BV) and challenged after one week by daily i.n. instillations of 10 µg HDM extract for 5 consecutive days as previously described (16). Control animals received the same volume of PBS. Mice were sacrificed 72 h after the last HDM challenge. Serum was prepared and stored at -80°C for IgE analysis. BALF was collected and differential cell counts were performed as previously described (34).

### Analysis of total IgE

Aliquots of serum were assayed in duplicates for total IgE using ELISA Kits (EMIGHE, Invitrogen, detection range 0.1-100 ng/ml) following manufacturer’s instructions.

### Histological evaluation

After BAL, the left lobe of the lung was removed, fixed in 4% buffered formalin and embedded in paraffin. 5 µm thick sections were stained with periodic acid–Schiff (PAS). Mucus hypersecretion and inflammatory cell infiltration were graded in a blinded fashion on a scale from 0 to 4, where 0=none; 0.5=very mild; 1=mild; 2=moderate; 3=marked and 4=severe as described in (34).

### Statistics

Data is displayed as mean ± SD. For statistical analysis, Student’s unpaired two-tailed t-test with Welch’s correction or Wilcoxon-Mann-Whitney test was performed using GraphPad Prism 8 (GraphPad Software Inc.). A p value of p < 0.05 was considered statistically significant.

## Results

### Bone marrow-derived macrophages reveal major AhR-dependent differences in gene clusters related to lipid mediator production

Myeloid cell types such as alveolar macrophages are known to synthesize and release a number of lipid mediators upon contact with LPS and allergens which play an important role in pathogenesis but also resolution of inflammation (12). We have previously shown that the absence of AhR triggers an aggravated inflammatory response in both pollen and HDM-induced AAI (16). In order to investigate whether alveolar-like macrophages might be compromised in their lipid mediator synthesis capacity, we differentiated BMDMs from wildtype and AhR-deficient animals in the presence of TGF-β to mimic alveolar-like macrophages (24) and stimulated them with the TLR4 ligand LPS. Bulk RNA sequencing of unstimulated- and LPS-stimulated BMDMs revealed major differences in gene expression profiles according to genotype (**Figure 1A**). Among other genes, AhR-deficient BMDMs showed reduced expression of *ALOX5*, *PTGS1* and *LTC4S* among the most differentially expressed genes (DEGs) between AhR-deficient and control BMDMs after LPS stimulation (**Figure 1A**, arrows). We then performed pathway analysis with the DEGs from these samples and noticed that pathways such as lipid metabolic pathways and tryptophan metabolism were significantly reduced in AhR-deficient BMDMs (**Figure 1B**). Besides external sources, tryptophan metabolism is known as the primary cell-intrinsic pathway to generate AhR ligands (35). Together, these data suggest that AhR activity may regulate lipid mediator synthesis in alveolar-like macrophages.

In parallel to RNA sequencing, we performed ATAC sequencing to investigate the chromatin accessibility and determined differential accessible regions (DARs). We then systematically compared gene expression and chromatin accessibility from the same samples (**Figure 1C**). 149 and 277 genes with increased expression (DEGup in **Figure 1C**) were associated to regions with increased chromatin accessibility (DARup) in unstimulated and LPS-stimulated AhR-deficient BMDMs, respectively. By contrast, 339 and 610 genes with decreased expression (DEGdown) were associated to regions with decreased chromatin accessibility (DARdown) in unstimulated and LPS-stimulated AhR-deficient BMDMs, respectively. Many more genes showed either selective regions with differential chromatin accessibility or gene expression profiles according to genotype. We therefore focused on a cluster of curated eicosanoid relevant genes (ERGs) (**Figure 1E**). Indeed, a cluster of these genes was significantly downregulated in AhR-deficient BMDMs (*ALOX5*, *LTC4S*, *HPGDS*, *PTGS1*) irrespective of whether BMDMs have been stimulated or not while few genes showed a stronger expression in AhR-deficient BMDMs upon LPS stimulation (*PTGIS*, *ALOX12*, *PTGS2*) (**Figure 1D**, black boxes). Interestingly, elevated induction of COX-2 expression (encoded by *PTGS2*) has already been observed in an AhR-deficient cell line of alveolar type 2 epithelial cells (17). We then searched for ERGs that showed differential expression and an association to differential chromatin accessibility and identified the enzymes 5-LO (*ALOX5*) and COX-1 (*PTGS1*) as two of the few genes fitting to this definition (**Figure 1E**). Overall, the mean normalized ATAC-seq coverage of downregulated DARs associated to differentially expressed ERGs (the intersection in **Figure 1E**) revealed a two- to threefold reduction in AhR-deficient BMDMs (**Figure 1F**). For instance, the *PTGS1* locus contains 4 intervals in which the AhR-deficient BMDMs displayed a significantly lower chromatin accessibility (**Figure 1G**). Importantly, one DAR associated to the *PTGS1* gene contained two AhR/Arnt motifs (**Figure 1G** inlet) suggesting a direct regulation of *PTGS1* expression by AhR. Thus, the AhR transcription factor regulates a number of genes involved in lipid mediator synthesis in part by regulating by chromatin accessibility.

### AhR-deficient macrophages have an altered lipid mediator profile

**Figure 2:**
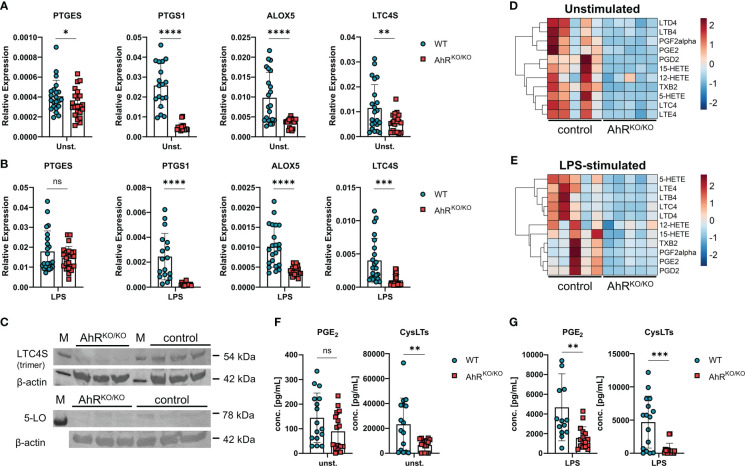
Impaired generation of prostanoids and cysteinyl leukotrienes in AhR-deficient macrophages. **(A)** Relative expression of *PTGES*, *PTGS1*, *ALOX5 and LTC4S* of unstimulated wildtype (WT) and AhR-deficient (AhR^KO/KO^) BMDMs. Data are derived from N = 22 animals per group from four independent experiments. **(B)** Relative expression of *PTGES*, *PTGS1*, *ALOX5 and LTC4S* of LPS-stimulated WT and AhR^KO/KO^ BMDMs. Data are derived from N = 22 animals per group from four independent experiments. **(C)** Western blot analysis of LTC4S (upper plot) and 5-LO (lower plot) of N = 3 biological triplicates of WT (control) and AhR^KO/KO^ BMDMs. Detection of β-actin in the same plot is shown below. **(D, E)** LC-MS/MS measurement of selected lipid mediators from BMDMs left either unstimulated or treated with LPS as in **(B)** Data are derived from N = 5 animals per group from two independent experiments. **(F, G)** Enzyme-linked immunosorbent (EIA) measurement for PGE_2_ (left plot) or total cysteinyl leukotrienes (cysLTs) (right plot) from unstimulated **(F)** or LPS-stimulated **(G)** WT and AhR^KO/KO^ BMDMs. Data are derived from N = 16 animals per group from four independent experiments. Each dot represents the result of an individual mouse and statistical significance was assessed with unpaired student’s t-test and p values of <0.05 were considered statistically significant. ns = not significant, *p<0.05, **p<0.01, ***p<0.001, ****p<0.0001.

Next, we wanted to confirm the expression patterns of enzymes involved in eicosanoid biosynthesis in AhR-sufficient and AhR-deficient BMDMs. Indeed, AhR-deficient BMDMs showed consistently reduced expression of the microsomal prostaglandin E Synthase-1 mPGES-1 (*PTGES*), COX-1 (*PTGS1*), 5-LO (*ALOX5*) and LTC4S (*LTC4S*) even in unstimulated cells compared to WT BMDMs (**Figure 2A**). As innate immune stimulatory agents such as LPS are often considered as one of the factors influencing the atopic potential during allergen exposure (36, 37) and we have previously observed enhanced allergic airway inflammation in AhR-deficient animals upon HDM exposure (16), we next tested whether LPS and HDM stimulation of BMDMs mitigates these AhR-dependent differences in gene expression. As expected, BMDM stimulation with LPS or HDM affected the expression of the aforementioned genes, but the AhR-dependent differences in gene expression were overall maintained (**Figure 2B**; **Supplementary Figure 1A**). Moreover, we confirmed higher levels of LTC4S and in tendency for 5-LO in LPS-stimulated BMDMs of WT mice by western blot analysis (**Figure 2C**, LTC4S detected as trimer (38, 39)). In order to test the functional consequences for eicosanoid production, we profiled these samples for a panel of 15 lipid mediators by liquid chromatography coupled to mass spectrometry (LC-MS/MS) after short term stimulation with ionophore to trigger maximal release of eicosanoids. Indeed, we found reduced amounts of prostanoids (PGD_2_, PGE_2_ and TBX_2_) and leukotrienes (LTB_4_, LTC_4_, LTD_4_ and LTE_4_) which was again obvious in unstimulated and LPS stimulated BMDMs (**Figures 2D, E**; complete results in **Supplementary Figure 1B**). Moreover, synthesis of the LOX-derived hydroxyeicosatetraenoic acids 5-HETE and 15-HETE was significantly lower in AhR-deficient BMDMs whereas 12-HETE was not affected (**Supplementary Figure 1B**), possibly due to compensatory upregulation of *ALOX12* (**Figure 1D**). The levels of arachidonic acid did not show a AhR-dependent profile suggesting that AhR regulates lipid mediator production in macrophages downstream of phospholipase A_2_ (**Supplementary Figure 1B**). Finally, we also quantified PGE_2_ and cysLT levels by an enzyme-linked immunosorbent assay. Indeed, cysLT levels were significantly reduced in unstimulated AhR-deficient BMDM cultures (**Figure 2F**). As expected, LPS and HDM stimulation of BMDMs strongly induced PGE_2_ production but similar to cysLTs, this response was greatly attenuated in AhR-deficient BMDMs (**Figure 2G**; **Supplementary Figure 1C**). Altogether, these results identify the AhR as a positive regulator of major prostanoids and leukotrienes in macrophages.

### Altered lipid mediator profile in AhR-dependent BMDMs is related to AhR activity and independent of stimulus

**Figure 3:**
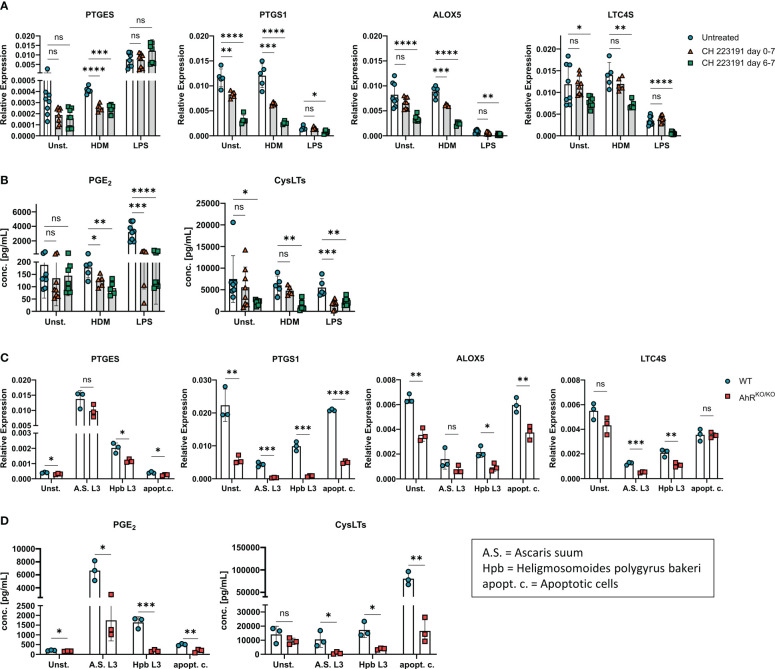
AhR inhibitor treatment recapitulates AhR-dependent effects on eicosanoids. **(A, B)** WT BMDMs were either treated just prior to HDM or LPS stimulation (day 6-7, green squares) or from day 0 onwards throughout BMDM differentiation (day 0-7, red triangles) with the AhR inhibitor CH-223191. Untreated WT BMDMs were used as control (blue circles). **(A)** Relative expression of *PTGES*, *PTGS1*, *ALOX5 and LTC4S* of unstimulated, HDM-stimulated or LPS-stimulated BMDMs are shown. **(B)** EIA measurement of PGE_2_ (left) and total cysLTs (right) from the samples in A. Data are derived from N = 5-8 animals per group from two independent experiments. **(C, D)** WT or AhR^KO/KO^ BMDMs were either unstimulated or stimulated with parasite extracts from *Ascaris suum* (A.S. L3), *Heligmosomoides polygyrus bakeri* (Hpb L3) or with apoptotic cells (apopt.c.), respectively. Relative expression of *PTGES*, *PTGS1*, *ALOX5 and LTC4S* and concentrations of PGE_2_ (left) and total cysLTs (right) **(D)** are shown. Data show pooled results of one representative experiment **(C, D)** with N = 3 animals per group. Each dot represents the result of an individual mouse and statistical significance was assessed with unpaired student’s t-test and p values of <0.05 were considered statistically significant. ns = not significant, *p<0.05, **p<0.01, ***p<0.001, ****p<0.0001.

So far, our results indicate a major role of the transcription factor AhR for the regulation of gene clusters associated to eicosanoid biosynthesis. In order to proof these findings and to exclude the possibility that the observed effect is due to altered myeloid differentiation in the absence of AhR (40), we employed the AhR antagonist CH-223191 to block AhR activity in wildtype BMDMs either just during restimulation with LPS or HDM (day 6) or throughout BMDM differentiation (day 0). Again, we found a modest reduction of *PTGES* expression in HDM stimulated BMDMs treated with the AhR antagonist which was not seen upon LPS stimulation (**Figure 3A**). However, *PTGS1* and *ALOX5* showed a reduced expression in CH-223191-treated cultures irrespective of whether BMDMs had been treated just prior to stimulation or throughout BMDM differentiation. For *LTC4S*, the expression pattern was more diverse and inhibition of AhR resulted only in a reduced expression when BMDMs were treated throughout differentiation (**Figure 3A**). AhR inhibitor treatment throughout the culture period also functionally affected eicosanoid biosynthesis because AhR antagonist treated BMDMs produced less PGE_2_ after HDM or LPS stimulation and reduced amounts of cysLTs already at baseline (**Figure 3B**). When BMDMs were treated with the AhR inhibitor just prior to stimulation on day 6, lower levels of PGE_2_ were released upon HDM or LPS stimulation whereas cysLTs were reduced only upon LPS stimulation (**Figure 3B**). Thus, blocking AhR activity by either using genetic AhR deficient cells (**Figure 2**) or by inhibiting AhR with a chemical antagonist (**Figure 3**) both resulted in a strong reduction of PGE_2_ and cysLTs synthesis in stimulated alveolar-like macrophages. Despite slight differences in the magnitude, AhR inhibition just prior to stimulation recapitulated the effect of AhR-deficient BMDMs suggesting a direct role for the AhR for the regulation of ERGs beyond possible effects on myeloid differentiation.

As LPS is typically contained in low amounts in HDM extracts, we next sought to determine whether other stimuli are able to evoke or maintain a similar AhR-dependent effect on PGE_2_ and cysLTs. To this end, we stimulated either wildtype or AhR-deficient BMDM cultures with parasites extracts of *Ascaris suum* (L3 larvae) or *Heligmosomoides polygyrus bakeri* (L3 larvae) both of which have been shown to influence lipid mediator production in macrophages (11, 41). Moreover, we also employed apoptotic cells as a stimulus because the uptake of apoptotic bodies by macrophages has been linked to activation of the AhR pathway (42). Stimulation of BMDMs with both parasite extracts maintained reduced *PTGS1* and *LTC4S* expression in AhR-deficient BMDMS, while only stimulation with Hpb extract resulted in reduced *PTGES* and *ALOX5* in AhR-deficient BMDMs when compared to WT BMDMs (**Figure 3C**). Stimulation of BMDMs with apoptotic cell bodies equally showed an AhR-dependent expression of *PTGES*, *PTGS1* and *ALOX5* (**Figure 3C**). Moreover, all stimulations resulted in a reduced synthesis of PGE_2_ and total cysLTs in AhR-deficient BMDMs (**Figure 3D**). Altogether, these results strongly suggest that the AhR is a major rheostat for macrophages’ ability to generate PGE_2_ and cysLTs. This ability is already compromised in unstimulated macrophages devoid of AhR signaling and amplified in activated macrophages but essentially independent of the macrophage’s activation signal.

### Alveolar macrophages show AhR-dependent regulation of eicosanoids *ex vivo*

**Figure 4:**
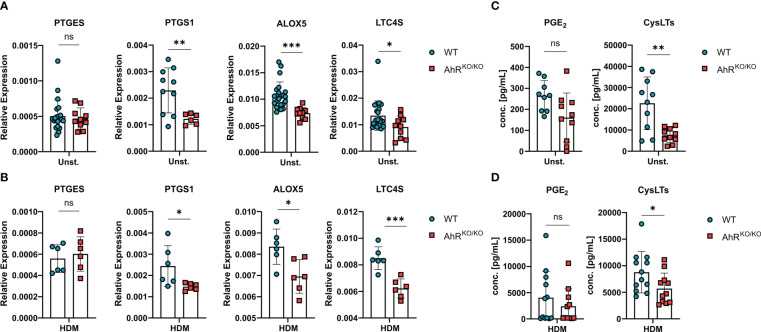
Eicosanoid production is also impaired in AhR-deficient alveolar macrophages. **(A, B)** Ex-vivo-isolated alveolar macrophages of either WT or AhR^KO/KO^ mice were either left untreated or stimulated with HDM and analyzed for gene expression and release of lipid mediators. Relative expression of *PTGES*, *PTGS1*, *ALOX5 and LTC4S* in alveolar macrophages with of the indicated genotype without **(A)** or after HDM stimulation **(B)**. Data are derived from N = 10-22 animals per group from five independent experiments. **(C, D)** PGE_2_ and total cysLTs levels of alveolar macrophages without **(C)** and with HDM stimulation **(D)**. Each dot represents the result of an individual mouse. Data show pooled results of two independent experiments with N = 12 animals per group. Each dot represents the result of an individual mouse and statistical significance was assessed with unpaired student’s t-test and p values of <0.05 were considered statistically significant. ns = not significant, *p<0.05, **p<0.01, ***p<0.001.

Even though BMDMs are a useful cellular tool to study basic properties of macrophages, they cannot fully recapitulate the differentiation and specific tissue adaptations of macrophages *in vivo*. Therefore, we sought to verify AhR-dependent regulation of eicosanoid biosynthesis directly in alveolar macrophages (**Figure 4A**). Noteworthy, *ex vivo*-isolated AhR-deficient alveolar macrophages readily showed reduced expression of *PTGS1*, *ALOX5* and *LTC4S* without stimulation (**Figure 4A**). This reduced expression pattern was maintained in AhR-deficient alveolar macrophages upon HDM stimulation suggesting a possible role for the AhR-eicosanoid axis of aeroallergen-stimulated macrophages (**Figure 4B**). Lastly, we also measured total PGE_2_ and cysLTs release from these cultures after ionophore treatment. While we observed a trend for reduced production of PGE_2_, AhR-deficient macrophages produced only half the amount of cysLTs in unstimulated cultures (**Figure 4C**). Even upon HDM stimulation AhR-deficient macrophages continued to produce significantly less cysLTs (**Figure 4D**). Altogether, these results suggest that AhR activity plays a regulatory role in the production of cysLTs also in primary alveolar macrophages.

### Hematopoietic expression of AhR is required to prevent exacerbation of HDM-induced AAI

**Figure 5:**
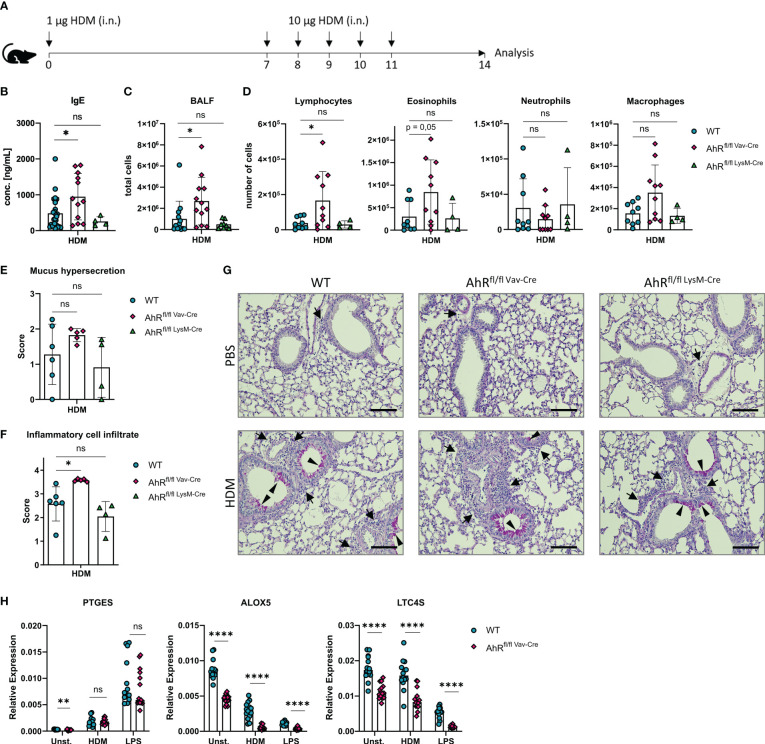
Hematopoietic AhR expression prevents exacerbation of allergic airway inflammation in a model of house dust mite allergy. **(A)** Experimental layout for HDM-induced allergic airway inflammation. **(B)** Serum IgE concentrations of AhR^flox/flox^ x Vav^Cre^, AhR^flox/flox^ x LysM^Cre^ or Cre-negative littermate control animals subjected to the protocol in A. Data are derived from N = 4-22 animals per group from at least two independent experiments. **(C)** Total cell counts in bronchoalveolar fluid (BALF). Data are derived from N = 8-13 animals per group from two to four independent experiments. **D**) Differential cell counts for lymphocytes, eosinophils, neutrophils and macrophages in BALF. Histological scores **(E, F)** and representative PAS stainings **(G)** from lung sections of PBS- or HDM-exposed mice of the indicated genotype. Arrows: inflammatory infiltrate; arrowheads: mucus hypersecretion; scale bar: 100µm. Data are derived from N = 4-6 representative animals per group from two independent experiments. **(H)** Relative expression of *PTGES*, *ALOX5 and LTC4S* in unstimulated, HDM- and LPS-treated BMDMs of AhR^flox/flox^ x Vav^Cre^ or control animals. Each dot represents the result of an individual mouse. Data show pooled results of two independent experiments with N = 13-15 animals per group. Each dot represents the result of an individual mouse and statistical significance was assessed with unpaired student’s t-test and p values of <0.05 were considered statistically significant. ns = not significant, *p<0.05, **p<0.01, ****p<0.0001.

The results outlined above and previous work from our groups indicate that HDM evokes robust eicosanoid production in alveolar macrophages (12, 43). Furthermore, monocyte-derived macrophages and alveolar macrophages of asthmatic individuals as well as alveolar-like BMDMs of HDM-challenged mice synthesize more cysLTs compared to cells from non-allergic controls (43, 44). We have previously shown that AhR-deficient animals show an exacerbated airway inflammation upon HDM challenge (16). Here, we sought to investigate whether AhR expression in macrophages is sufficient to prevent this exacerbation of the allergic response. Therefore, we crossed LysM^Cre^ and Vav^Cre^ mice with animals harboring floxed alleles of exon 2 of the AhR gene to ablate AhR expression in either LysM-expressing macrophages, monocytes and neutrophils (LysM^Cre^) or all hematopoietic cells (Vav^Cre^), respectively, and subjected these animals to an established HDM model to elicit allergic airway inflammation (**Figure 5A**). Interestingly, Vav^Cre^ x AhR^flox/flox^ animals but not LysM^Cre^ x AhR^flox/flox^ animals showed elevated IgE levels (**Figure 5B**), elevated total cell counts in the bronchoalveolar lavage (BALF) (**Figure 5C**) and higher lymphocyte and eosinophil cell counts when compared to control animals (**Figure 5D**). Strikingly, LysM^Cre^ x AhR^flox/flox^ animals did not show this pattern which prompted us to investigate the recombination efficiency in alveolar-like BMDMs of these animals. Moreover, histological analysis of HDM-treated Vav^Cre^ x AhR^flox/flox^ animals revealed a stronger cellular infiltration and a tendency for enhanced mucus production (**Figures 5E–G**). To investigate potential differences in recombination efficiencies of Vav^Cre^ and LysM^Cre^ lines, we generated again alveolar-like BMDMs and measured *AhR* expression. As expected, *AhR* expression could not be detected in BMDMs of full AhR-deficient or Vav^Cre^ x AhR^flox/flox^ animals (**Supplementary Figure 2A**). However, alveolar-like BMDMs of LysM^Cre^ x AhR^flox/flox^ animals showed only a reduction of AhR expression by approximately 80%. Moreover, the expression of *PTGES*, *ALOX5* and *LTC4S* did not reveal a comparable reduction in expression levels compared to full AhR-knockout BMDMs (**Supplementary Figure 2B**). Finally, we stimulated BMDMs of Vav^Cre^ x AhR^flox/flox^ animals with HDM and LPS and measured the expression of *PTGES*, *ALOX5* and *LTC4S*. We could confirm lower expression of *ALOX*5 and *LTC4S* in the absence of hematopoietic AhR expression while reduced expression of *PTGES* was only visible in unstimulated cells (**Figure 5H**).

To rule out an AhR-dependent contribution of lung epithelial cells to the exacerbation of the allergic airway inflammation, we crossed Scgb1a1^CreERT2^ animals to AhR^flox/flox^ animals. The corresponding offspring was treated from week 3 onwards with tamoxifen to ablate AhR expression in all Scgb1a1^+^ lung epithelial cells and their progenies and subjected tamoxifen-treated mice to the HDM model (**Supplementary Figure 3A**). Importantly, we could not detect any difference in BALF cell counts or IgE levels between tamoxifen-treated Scgb1a1^CreERT2^ x AhR^flox/flox^ and their littermate controls, suggesting that AhR expression in lung epithelial cells is not required for preventing exacerbated inflammation (**Supplementary Figures 3B–D**). Altogether, these results strongly argue for an important function of AhR activity in alveolar macrophages for the regulation of HDM-induced allergic airway inflammation. The AhR-dependent transcriptional regulation of prostanoid and cysLT synthesis likely plays an important role in governing allergic inflammation of the airways.

## Discussion

In this study we investigated whether the transcription factor AhR affects arachidonic acid-derived lipid mediator biosynthesis in macrophages as a possible explanation for enhanced susceptibility of AhR-deficient animals to allergic airway inflammation (16). Our results demonstrate that AhR-deficient alveolar and bone marrow-derived alveolar-like macrophages showed a systematically reduced production of prostanoids and cysLTs across a range of different stimuli including allergens. This AhR-dependent regulation of eicosanoid synthesis was even detectable in resting macrophages and could be confirmed in WT macrophages treated with an AhR antagonist, excluding the possibility that a reduced capacity to synthesize eicosanoids is a side effect of gene targeting in AhR-deficient macrophages. More generally, bulk RNA and ATAC sequencing of alveolar-like macrophages revealed that AhR regulates a set of genes involved in rate-limiting steps of eicosanoid biosynthesis. For instance, AhR-deficient macrophages showed reduced expression of *LTC4S* and *ALOX5*. 5-LO, the product of *ALOX5*, regulates the formation of LTA_4_ which is further metabolized *via* the activity of LTC4S to LTC_4_ and subsequently to LTD_4_ and LTE_4_. The formation of LTB_4_ does not depend directly on LTC4S activity but is dependent on LTA_4_ levels. Our data clearly suggest that AhR is a transcriptional regulator for 5-LO and LTC4S resulting in a reduced capability of AhR-deficient macrophages to generate cysLTs. Within the prostanoid pathway, we initially focused on a possible transcriptional regulation of mPGES-1 as the rate-limiting enzyme for the generation of anti-inflammatory PGE_2_ in type 2 immunity (7, 8) because reduced levels of PGE_2_ may contribute to an increased allergic airway inflammation in the absence of AhR activity (16). PGE_2_ is synthesized from PGH_2_ *via* mPGES-1 activity but, surprisingly, also PGD_2_ levels were reduced in AhR-deficient BMDMs (**Figures 2D, E**). This prompted us to investigate also the expression of the more upstream COX enzymes. Noteworthy, both bulk sequencing and quantitative analysis revealed marked reduction of COX1 expression (encoded by *PTGS1*). Both COX-1 and COX-2 isoenzymes can convert AA into PGH_2_ but only COX2 expression is typically upregulated during inflammation while COX1 is thought to be expressed in a more constitutive manner and fulfils basic physiological functions in both immune and non-immune cell types (45). A reduced expression of COX-1 may thus suggest that the basic capacity of AhR-deficient macrophages to generate prostanoids is compromised. In contrast to COX-1, COX-2 expression could be upregulated more strongly in AhR-deficient BMDMs (**Figures 1A, D**), a phenomenon that has also been observed in an AhR-deficient alveolar type II cell line (17). Yet, this compensatory upregulation of COX-2 was not sufficient to overcome reduced COX-1 activity and the physiological generation of prostanoids.

AhR deficiency may also compromise the differentiation of myeloid cells including alveolar-like macrophages that may have an important long-term effect on airway pathogenesis. Indeed, AhR activity has been linked to the differentiation of monocytes towards the dendritic cell versus the macrophage lineage (40) and the basic capability for eicosanoid biosynthesis differs between both cell types (46, 47). However, as AhR inhibitor treatment of WT cells after BMDM differentiation was equally able to reduce the expression of COX-1 (*PTGS1*) and 5-LO (*ALOX5*) and partially also reduce LCT4S and mPGES-1 expression, we propose that AhR activity regulates the expression of these enzymes independently of potential AhR effects on myeloid differentiation. During allergic responses, the reduced COX-1-driven regulation by anti-inflammatory prostanoids such as PGE_2_ in the absence of AhR activity cannot compensate the reduced synthesis of pro-inflammatory cysLTs which would normally be expected to result in reduced inflammation. Instead, as both rate-limiting enzymes of the prostanoid and leukotriene pathway (5-LO and COX-1) are compromised in the absence of AhR activity, AhR signaling in macrophages may be required to engage the full potential of alveolar macrophages for the generation of eicosanoids. In the absence of this potential, the regulatory role of eicosanoids is vanished and associated with an enhanced risk for uncontrolled allergic sensitization and inflammation. Importantly, the AhR has been previously shown to act as a break for the release of pro-inflammatory cytokines upon stimulation of macrophages with LPS (48–51). However, this hypersensitivity and the aggravated inflammation observed *in vivo* often prevents to dissect the underlying molecular mechanisms in a single cell type given the manifold roles the AhR can play in different immune cells.

Along this hypothesis, we found that hematopoietic but not epithelial AhR deficiency recapitulated exaggerated inflammation in the HDM model (**Figure 5**; **Supplementary Figure 3**) which we have observed previously in complete AhR deficient animals (16). One limitation of our study is the possible contribution of other hematopoietic cell types to the exaggerated inflammation such as dendritic cells or granulocytes. In this regard, the LysM^Cre^ mouse line may not be a suitable Cre-expressing line to ablate AhR gene expression with high efficiency in alveolar macrophages. Additionally, minimal AhR expression may be sufficient for AhR-dependent regulation of eicosanoid biosynthesis relevant genes while targeting other genes using the LysM^Cre^ line may show a stronger dose-dependence in alveolar macrophages.

Unfortunately, the profiling of eicosanoids in bronchoalveolar fluids of HDM-treated animals by LC-MS/MS was not successful as eicosanoid levels in many samples were below the lower limit of detection (not shown). However, macrophages are thought to constitute one of the major eicosanoid-producing cell type and in contrast to intestinal eosinophils, pulmonary eosinophils have so far not been shown to be regulated by AhR activity (52).

Whether AhR ligands are able to foster the generation of either prostanoid or cysLT production in a ligand-specific manner and may thus represent a suitable therapeutic strategy will be part of future investigations. As environmental pollutants often contain AhR ligands acting either as agonists or antagonists (53), AhR-dependent regulation of bioactive lipid mediators may also provide a novel basis to explore AhR pollutants in epidemiological studies. Indeed, systemic activation by the potent AhR agonist 2,3,7,8-Tetrachlorodibenzo-p-dioxin (TCDD) revealed an alteration in arachidonic acid metabolism and eicosanoid biosynthesis including different expression levels of 5-LO and LTC4S and an increase of pro-inflammatory leukotrienes in the liver (54, 55). Whether these observations are related to AhR-dependent gene regulation in Kupfer cells or hepatocytes remains to be determined in future studies. Noteworthy, the AhR immediate target enzymes of the cytochrome P450 CYP1 family may also regulate bioavailability of eicosanoids because these enzymes are able to oxidize and degrade lipid mediators (56, 57). Noteworthy, we did not supply any specific AhR ligand to our cultures suggesting that the AhR-eicosanoid axis is active without external AhR ligands and fueled by cell-intrinsic ligands.

Eicosanoids play a fundamental role in a variety of physiological processes and diseases beyond allergic diseases. With regard to possible translation of these findings for humans, it is therefore necessary to investigate whether first, human macrophages exhibit a similar AhR dependency for eicosanoid biosynthesis and second, whether this axis is disturbed in patients with type 2 immune disorders, such as allergic asthma and related diseases. Whether impaired AhR activity and associated reduced generation of anti-inflammatory lipid mediators or conversely, excessive AhR activity and associated excessive release of pro-inflammatory lipid mediators contribute to disease pathogenesis remains an interesting study goal and may be one avenue to link the environmental sensor AhR to dysregulated type 2 immune responses at barrier organs.

## Data availability statement

The datasets presented in this study can be found in online repositories. The names of the repository/repositories and accession number(s) can be found below: GSE221093 and GSE221144 (GEO).

## Ethics statement

The animal study was reviewed and approved by local ethics committee and government authorities (Upper Bavaria) under the numbers ROB-55.2-2532.Vet_02-18-94 and ROB-55.2-2532.Vet_02-17-222.

## Author contributions

A-MM performed most experiments and data analysis. KH, FA, BS and AA contributed to experiments and analysis. FH, FR and MH performed LC-MS/MS measurements. AK and KS performed bioinformatic analysis. CS-W and JE-B cosupervised the study. CO supervised the study, performed data interpretation, acquired funding and wrote the manuscript with input from all co-authors. All authors contributed to the article and approved the submitted version.
