# Corrigendum: The aryl hydrocarbon receptor regulates lipid mediator production in alveolar macrophages

**DOI:** 10.3389/fimmu.2023.1221064

**Published:** 2023-06-05

**Authors:** Ann-Marie Maier, Karsten Huth, Francesca Alessandrini, Fiona Henkel, Benjamin Schnautz, Anela Arifovic, Fabien Riols, Mark Haid, Anja Koegler, Katrin Sameith, Carsten B. Schmidt-Weber, Julia Esser-von-Bieren, Caspar Ohnmacht

**Affiliations:** ^1^ Center of Allergy and Environment (ZAUM), Technical University of Munich and Helmholtz Center Munich, Research Center for Environmental Health, Neuherberg, Germany; ^2^ Metabolomics and Proteomics Core, Helmholtz Center Munich, Research Center for Environmental Health, Neuherberg, Germany; ^3^ DRESDEN-concept Genome Center, Technology Platform at the Center for Molecular and Cellular Bioengineering (CMCB), Technische Universität Dresden, Dresden, Germany; ^4^ Member of the German Center of Lung Research (DZL), Partner Site Munich, Munich, Germany; ^5^ Department of Immunobiology, University of Lausanne, Epalinges, Switzerland

**Keywords:** macrophage, aryl hydrocarbon receptor, eicosanoids, leukotriene, prostaglandin

In the published article, there was an error in the author list, and author Fiona Henkel was erroneously excluded. The corrected author list appears below.

Ann-Marie Maier^1^, Karsten Huth^1^, Francesca Alessandrini^1^, Fiona Henkel^1^, Benjamin Schnautz^1^, Anela Arifovic^1^, Fabien Riols^2^, Mark Haid^2^, Anja Koegler^3^, Katrin Sameith^3^, Carsten B Schmidt-Weber^1,4^, Julia Esser-von-Bieren^1,5^, Caspar Ohnmacht^1^

Fiona Henkel is therefore removed from the Acknowledgments. The corrected Acknowledgments appears below.

We thank Johanna Grosch for help with the preparation of histology slides.

New author Fiona Henkel is now part of the Author contributions. The corrected Author contributions statement appears below.

A-MM performed most experiments and data analysis. KH, FA, BS and AA contributed to experiments and analysis. FH, FR and MH performed LC-MS/MS measurements. AK and KS performed bioinformatic analysis. CS-W and JE-B co-supervised the study. CO supervised the study, performed data interpretation, acquired funding and wrote the manuscript with input from all co-authors. All authors contributed to the article and approved the submitted version.

The authors apologize for this error and state that this does not change the scientific conclusions of the article in any way. The original article has been updated.

