# Supplementary material for: The aryl hydrocarbon receptor regulates lipid mediator production in alveolar macrophages
Source: Front Immunol. 2023 Apr 4;14:1157373. doi: 10.3389/fimmu.2023.1157373 (PMC10110899; doi:10.3389/fimmu.2023.1157373)
Supplement: Supplementary file 1 [file DataSheet_1.pdf]

Supplementary Figure 1

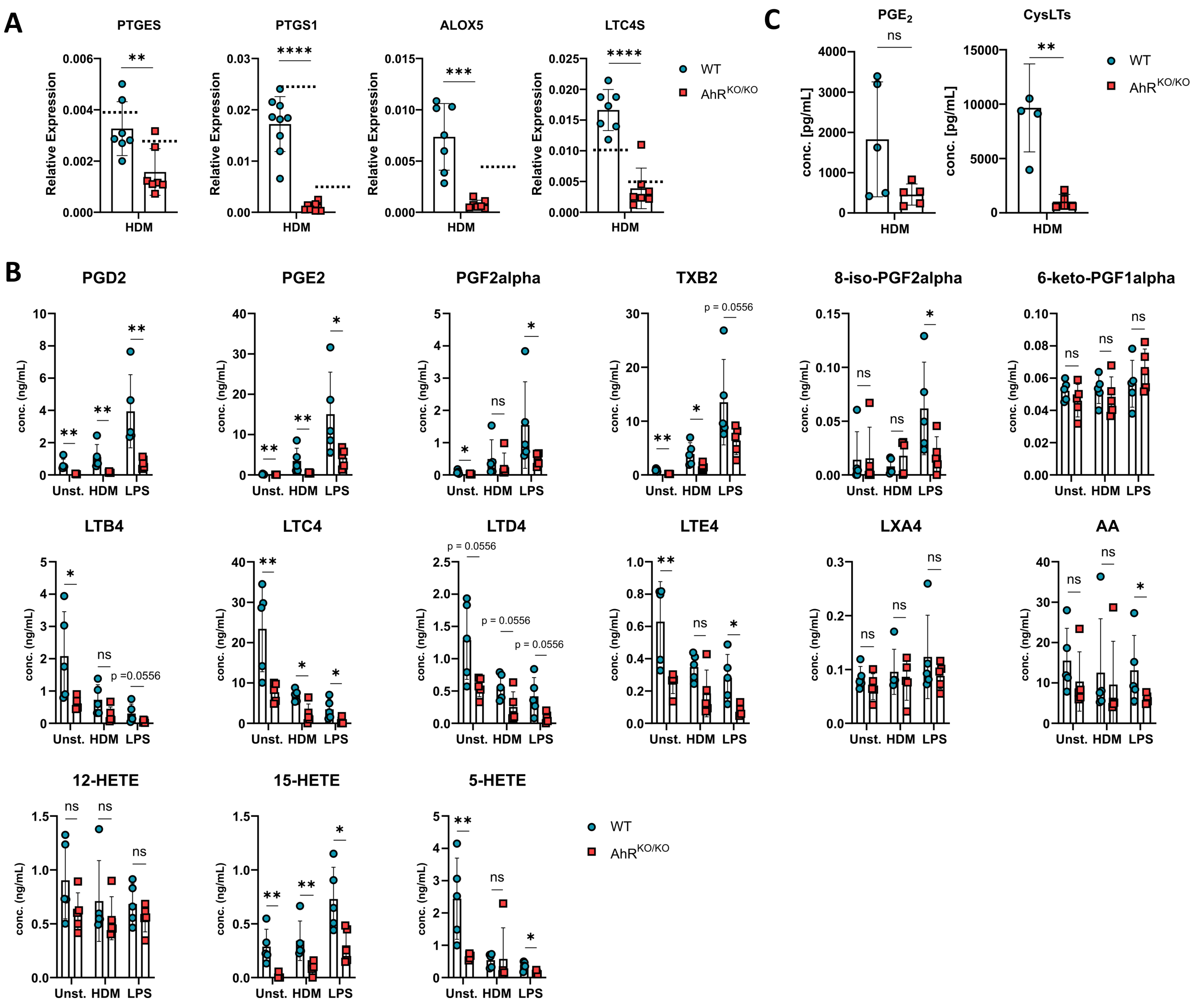

Supplementary Figure 1

AhR is a major regulator of prostanoid and leukotriene synthesis in alveolar-like macrophages

A) Relative expression of *PTGES*, *PTGS1*, *ALOX5 and LTC4S* of HDM-stimulated wildtype (WT) and AhR-deficient (AhR$^{KO/KO}$) BMDMs. Dotted lines indicate relative expression of unstimulated BMDMs. Data are derived from N = 7-9 animals per group from five independent experiments. **B)** LC-MS/MS measurement of indicated lipid mediators and arachidonic acid from BMDMs left either unstimulated or stimulated with HDM or LPS. Data are derived from N = 5 animals per group from two independent experiments. **C)** Enzyme-linked immunosorbent (EIA) measurement for PGE_2 (left plot) or total cysteinyl leukotrienes (cysLTs) (right plot) from HDM-stimulated WT and AhR$^{KO/KO}$ BMDMs. N = 4-5 animals per group from two independent experiments. Each dot represents the result of an individual mouse and statistical significance was assessed with unpaired student's t-test (A) or Mann-Whitney test (B and C) and p values of <0.05 were considered statistically significant. ns = not significant, * p<0.05, ** p<0.01, *** p<0.001, **** p<0.0001

Supplementary Figure 2

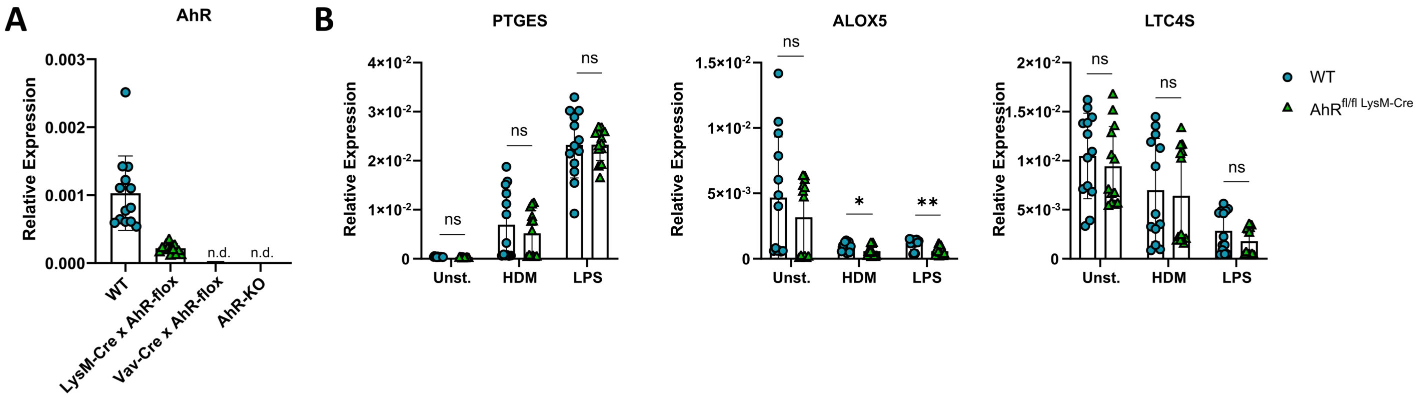

Supplementary Figure 2

LysM^{Cre} driven ablation of AhR is not efficient and does not affect gene expression related to eicosanoid biosynthesis

A) Relative *AhR* expression in BMDMs of AhR$^{KO/KO}$, AhR$^{flox/flox}$ x Vav^{Cre}, AhR$^{flox/flox}$ x LysM^{Cre} or control animals. Data are derived from N = 13-14 animals per group from two independent experiments. **B**) Relative expression of *PTGES*, *ALOX5 and LTC4S* in unstimulated, HDM- and LPS-treated BMDMs of the indicated genotypes. Data are derived from N = 13 animals per group from three independent experiments. Each dot represents the result of an individual mouse and statistical significance was assessed with unpaired student's t-test and p values of <0.05 were considered statistically significant. ns = not significant, * p<0.05, ** p<0.01

Supplementary Figure 3

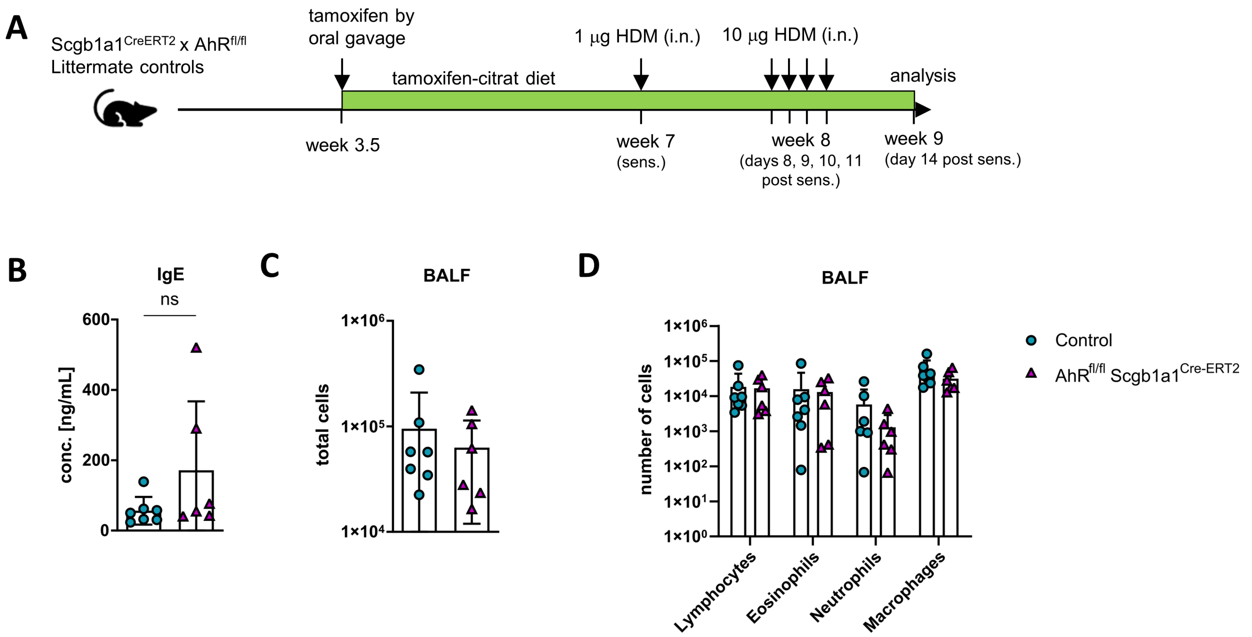

Supplementary Figure 3

Lung epithelial cell-specific ablation of AhR does not exacerbate house dust mite induced allergic airway inflammation

A) Experimental layout. **B**) Serum IgE concentrations of tamoxifen-treated $AhR^{flox/flox}$ x $Scgb1a1^{CreERT2}$ or Cre-negative littermate control animals subjected to the protocol in Fig. 5A. Data are derived from N = 6-7 animals per group from one representative experiment. **C**) Total cell counts in bronchoalveolar fluid (BALF) Data are derived from N = 6-7 animals per group from one representative experiment. **D**) Differential cell counts for lymphocytes, eosinophils, neutrophils and macrophages in BALF. Data are derived from N = 6-7 animals per group from one representative experiment. Each dot represents the result of an individual mouse and statistical significance was assessed with unpaired student's t-test and p values of <0.05 were considered statistically significant. ns = not significant
