# Supplementary material for: The aryl hydrocarbon receptor regulates lipid mediator production in alveolar macrophages
Source: Front Immunol. 2023 Apr 4;14:1157373. doi: 10.3389/fimmu.2023.1157373 (PMC10110899; doi:10.3389/fimmu.2023.1157373)
Supplement: Supplementary file 2 [file Table_1.docx]

**Forward and reverse primers for qPCR**

| **Gene** | **Forward Primer (5’ - 3’)** | **Reverse Primer (5’ - 3’)** |
| --- | --- | --- |
| AhR | TCTGTTCTTAGGCTCAGCGTC | GCGCCTGTAACAAGAACTCTC |
| ALOX5 | ATTGCCATCCAGCTCAACCA | ACTGGAACGCACCCAGATTT |
| GAPDH | CGTCCCGTAGACAAAATGGT | TTGATGGCAACAATCTCCAC |
| LTC4S | ATCTTCTTCCACGAAGGAGCC | TCGCGTATAGGGGAGTCAGC |
| PTGES | GAGTACACGAAGCCGAGGAA | GAAGAAGGCTTTTGCCAACC |
| PTGS1 | GGGAATTTGTGAATGCCACC | GGGATAAGGTTGGACCGCA |
| ß-Actin | TTCTTTGCAGCTCCTTCGTT | ATGGAGGGGAATACAGCCC |
